# Maternal Organic Selenium Supplementation Relieves Intestinal Endoplasmic Reticulum Stress in Piglets by Enhancing the Expression of Glutathione Peroxidase 4 and Selenoprotein S

**DOI:** 10.3389/fnut.2022.900421

**Published:** 2022-05-06

**Authors:** Dajiang Ding, Daolin Mou, Heng Zhu, Xuemei Jiang, Lianqiang Che, Zhengfeng Fang, Shengyu Xu, Yan Lin, Yong Zhuo, Jian Li, Chao Huang, Yuanfeng Zou, Lixia Li, De Wu, Bin Feng

**Affiliations:** ^1^Animal Nutrition Institute, Sichuan Agricultural University, Chengdu, China; ^2^Key Laboratory of Animal Disease-Resistant Nutrition of Ministry of Education, Sichuan Agricultural University, Chengdu, China; ^3^Key Laboratory of Animal Disease-Resistant Nutrition of Sichuan Province, Sichuan Agricultural University, Chengdu, China; ^4^College of Bioengineering, Sichuan University of Science and Engineering, Zigong, China; ^5^College of Veterinary Medicine, Sichuan Agricultural University, Chengdu, China

**Keywords:** selenium, ER stress, selenoprotein S, GPX4, intestine

## Abstract

Endoplasmic reticulum (ER) stress, which can be induced by reactive oxygen species (ROS) and multiple factors, is associated with numerous intestinal diseases. The organic selenium source 2-hydroxy-4-methylselenobutanoic acid (HMSeBA), has been proved to decrease intestinal inflammation and autophagy by improving the expression of selenoproteins. However, it remains unclear whether HMSeBA could alleviate intestinal ER stress by decreasing excessive production of ROS products. This study was conducted to investigate the effect of maternal HMSeBA supplementation on the regulation of intestinal ER stress of their offspring and the regulatory mechanism. Sows were supplemented with HMSeBA during gestation and jejunal epithelial (IPEC-J2) cells were treatment with HMSeBA. Results showed that maternal HMSeBA supplementation significantly upregulated mRNA level of selenoprotein S (SELS) in the jejunum of newborn and weaned piglets compared with the control group, while decreased the gene expression and protein abundance of ER stress markers in the jejunum of LPS challenged weaned piglets. In addition, HMSeBA treatment significantly increased the expression of glutathione peroxidase 4 (GPX4) and SELS, while decreased ROS level and the expression of ER stress markers induced by hydrogen peroxide (H_2_O_2_) in IPEC-J2 cells. Furthermore, knockdown of GPX4 did not enhance the ERS signal induced by H_2_O_2_, but the lack of GPX4 would cause further deterioration of ER stress signal in the absence of SELS. In conclusion, maternal HMSeBA supplementation might alleviate ROS induced intestinal ER stress by improving the expression of SELS and GPX4 in their offspring. Thus, maternal HMSeBA supplementation might be benefit for the intestinal health of their offspring.

## Introduction

Endoplasmic reticulum (ER) stress, which is well known as the accumulation of unfolded proteins in ER lumen caused by the increase of protein synthesis or multiple conditions, and ER responds to ER stress by activating the unfolded protein response (UPR), an adaptive intracellular signal to cope with ER stress and help to sustain cell survival and normal functions (1). Notably, the activation of UPR is intimately related to three transmembrane ER-resident stress sensors: inositol-requiring enzyme 1 (IRE1), PKR-like ER kinase (PERK) and activating transcription factor (ATF6), which acts as regulators of cell fate under ER stress by integrating information about the intensity and duration of the injury. These three ER stress sensors were binding to glucose-regulated protein 78 (GRP78) when below the threshold of UPR signal, thereby inhibiting the activation of UPR (2, 3). While all three branches may induce apoptotic cell death via upregulating the expression of the C/EBP homologous protein (CHOP) when UPR signal is continuously activated (4). Simultaneously, endoplasmic reticulum oxidoreductase 1 alpha (ERO1α) and endoplasmic reticulum oxidoreductase 1 beta (ERO1β), as the targets of CHOP, play the roles of generating ROS production and induce organ injury (5). It’s worth noting that, physiological stimuli, such as ROS and intestinal endogenous pathogenic microorganism product lipopolysaccharide (LPS) can also activate ERS *in vitro* and vivo (6–10).

The small intestinal, which plays roles in nutrient digestion and absorption, and also acts as an innate barrier against luminal pathogens and physiological stress. Intestinal redox homeostasis is a prerequisite for maintaining its function, while accumulation of reactive oxygen species (ROS) is the main cause of intestinal redox disorder induced by invasions of pathogenic microorganisms and various stimulates. As it mentioned above, ROS accumulation may cause ER stress (6, 7, 9), so it is not surprising that ER stress and the UPR signal is essential for shaping intestinal redox homeostasis. Indeed, the UPR has been regarded as an important pharmacological target in the development of therapeutic strategies for immune-mediated pathology (11). Numerous studies also proved that ER stress contributes to intestinal inflammation diseases via the activation of IRE1/ATF6-XBP1 signaling pathway (12–14). Thus, regulation of ER stress signal is essential for intestinal homeostasis and intestinal health.

Selenium is an essential trace element and the function of selenium is mediated in part by its incorporation into slenoproteins, and most of these selenoproteins have antioxidant properties such as glutathione peroxidase (GPX) and selenoprotein P (SEPP1) (15). Our previous study demonstrated that maternal HMSeBA (2-Hydroxy-4-methylselenobutanoic acid, a novel organic Se source) supplementation was beneficial for offspring’s intestinal health by regulating inflammation and autophagy via MAPK/NF-κB and ERK/Beclin-1 signaling pathways (16), while whether HMSeBA is involved in regulating ER stress signal of intestinal remains unclear. Moreover, numerous studies have proved from both pros and cons that SELS is important for regulating ER stress induced apoptosis by participating in unfolded and misfolded proteins degradation (17–19), and our study revealed that maternal HMSeBA supplementation could protect thymus and spleen against inflammation via inhibiting the ERS signal by improving the expression of GPX1, GPX4 and SEPP1 (20), whether these four selenoproteins participate the regulation of ER stress in intestinal also remains unclear. Besides, previous studies reached conclusions based on maternal effects of HMSeBA *in vivo* experiments, the direct regulation of HMSeBA on ER stress signal to porcine intestinal epithelial cells (IPEC-J2) also needs further study. In short, this study was conducted to investigate the protective effect of maternal HMSeBA supplementation in regulating ER stress signal induced by LPS in the jejunum of offspring, and the direct regulation of HMSeBA on ER stress signal induced by ROS and the expression of selenoproteins in IPEC-J2 cells. This study will systematically explained the synergistic effect of several selenoproteins in the intestine to regulate the ER stress from *in vivo* to *in vitro*, which provides more evidence for selenium or selenoproteins to protect intestinal health.

## Materials and Methods

### Animal Model

The experimental design of sows was described as previous study (21). Thirty sows (Landrace × Yorkshire) at 5th parity were randomly assigned into two groups to receive basal diet (control, *n* = 15), or HMSeBA supplemented diet (HMSeBA, basal diet + HMSeBA at 0.3 mg Se per kg, *n* = 15) during the whole gestation. HMSeBA was provided by Adisseo France S.A.S. and all diets were formulated to meet nutrient requirements of the National Research Council (22). After farrowing, the number of litters were adjusted to 12 ± 1 piglets per litter by cross-fostering in the same treatment and one piglet with body weight (BW) closed to average litter BW was selected to be slaughtered for jejunum collection within 24 h. Piglets were breast fed during lactation. At weaning, two piglets of each litter with BW closed to average litter BW were selected to be challenged with lipopolysaccharide (LPS, L2880, Sigma) at the dosage of 50 μg/kg BW or equivalent amount of sterile saline for 4 h. Piglets were then slaughtered for jejunum collection. All animals had free access to water.

### Cell Culture and Treatment

IPEC-J2 cells were obtained from ATCC and cultured in cell culture flasks or plates (Nunc.) with DMEM-F12 (Gibco, Grand Island, NY, United States) supplementing 10% FBS, penicillin (100 U/mL), and streptomycin (100 μg/mL), at 37°C in a 5% CO_2_ atmosphere humidified incubator. When the density reached about 70%, cells were pre-treated with different concentrations of HMSeBA (300, 600, and 1200 nM) for 24 h, attended by co-treatment with H_2_O_2_ (0.1, 0.2, and 0.4 mM) (Chron chemicals, Chengdu, China) for another 2 h.

### RNA Extraction and Real-Time Polymerase Chain Reaction Assay

Total RNA was extracted from frozen jejunum samples with TRIzol reagent (Sigma-Aldrich), and was used to synthesize cDNA with Prime-Script RT reagent kit (Takara, Dalian, China) according to the manufacturer’s instructions. Real-time PCR protocol was 1 cycle of 95°C for 30 s and 40 cycles of 95°C for 15 s followed by 60°C for 1 min. 2^–delta CT^ method was utilized to calculate the relative gene expression of target genes and β-actin were used as reference genes. Primers for the genes are listed in Supplementary Table 1.

### Western Blot Analysis

Jejunum tissues or cell samples were lysed with radioimmunoprecipitation assay (RIPA) lysis buffer (Beyotime, Shanghai, China) as previous report (16). The protein concentration was determined with bicinchoninic acid (BCA) protein assay kit (Thermo Scientific, Waltham, MA, United States.). 30 μg protein of each sample was separated in 10% (SDS-PAGE) gels (Beyotime, Shanghai, China). Proteins were subsequently transferred to PVDF membrane (Bio-Rad). The PVDF membrane was then blocked with 1% BSA containing TBST, followed by incubation with primary antibodies at 4°C for more than 8 h. The primary antibodies were as follows: SELS (21 KDa, 15591-1-AP, Proteintech), GPX4 (22 KDa, 52455S, Cell Signaling Technology), SEPP1 (57/45 KDa, sc-376858, Santa Cruz Biotechnology), GRP78 (78 KDa, ab21685, Abcam), p-IRE1α (110 KDa, ab48187, Abcam), IRE1α (110 KDa, ab37073, Abcam), p-PERK (170 KDa, 3179S, Cell Signaling Technology), PERK (170 KDa, 3192S, Cell Signaling Technology), CHOP (27 KDa, 381679, Zen BioScience) and β-actin (45 kDa, #4967, Cell Signaling Technology). The membrane was washed with TBST for 6 times, and incubated with appropriate HRP-conjugated secondary antibodies at room temperature for 1 h. After another 6-time wash, membrane was incubated with enhanced chemiluminescence (ECL) (Beyotime, Shanghai, China), and protein signals were accomplished with a ChemiDocTM XRS + Imager System (Bio-Rad Laboratories, Inc.).

### Analysis of Oxidant and Antioxidant Contents

Jejunum samples were rapidly weighted, homogenized and centrifuged. The supernatants were used to measure oxidant and antioxidant contents. The activities of glutathione peroxidase (GPH-Px), total superoxide dismutase (T-SOD), catalase (CAT), total antioxidant capacity (T-AOC) and contents of malondialdehyde (MDA) and hydrogen peroxide (H_2_O_2_) were quantified using the respective assay kits Nanjing Institute of Jiancheng Biological Engineering, Nanjing, China, according to the manufacturer’s protocols.

### Quantification of Reactive Oxygen Species

For quantification of ROS, when the density reached about 85%, IPEC-J2 cells were washed with PBS for 2 times, and then 5 μM 2′,7′-dichlorofluorescein diacetate (DCF-DA) reagent (Merck; 35845) was incubated with cells for 25 min at 37°C in the dark, finally, analyzed with flow cytometry after three times wash with PBS.

### Construction of Adenovirus and Gene Knockdown Study

To construct adenoviral vectors expressing short hairpin interfering RNAs (shRNAs) against pig GPX4 or SELS, three short hairpin oligonucleotides and complementary strands were designed to target pig GPX4 and SELS, respectively. Briefly, the top and bottom strand oligonucleotides were annealed and ligated into the Gateway-based pENTR/U6 vector (Thermofisher) and sequence confirmed. Then pENTR/U6-shRNA plasmids were recombined with the Gateway based pAd-BLOCK-iT DEST vector (Thermofisher) to generate pAd shRNA vector. The pAd shRNA plasmids were then transfected into HEK 293A cells (Invitrogen, Carlsbad, CA, United States) to generate adenovirus, after *P*ac I digestion. Adenovirus were then amplified HEK 293A cell, and the knockdown efficiency of the shRNA were examined in IPEC-J2 cells with multiplicity of infection (MOI) of 50 (Supplementary Figure 2). The sequence of shRNAs used in this study were GGAATTCTCAGCCAAGGACATC for GPX4 and GGAACCTGATGTTGTTGTTAA for SELS. Sequence of GCTACACAAATCAGCGATTT for shLacz was used as the control. For gene knockdown study, IPEC-J2 cells were infected with shLacz cloned adenovirus (AdshLacz) or shGPX4 cloned adenovirus (AdshGPX4) or shSELS cloned adenovirus (AdshSELS) at MOI of 50 for 48 h.

### Statistical Analyses

All data were analyzed with SPSS 27.0 (IBM SPSS Company, Chicago, IL, United States). Data of newborn piglets and cells were analyzed using two-tailed *t*-test. Data of weaned piglets after LPS challenge and cells treated with HMSeBA and H_2_O_2_ were performed with Tukey’s multiple comparisons using general linear model (GLM) procedure of SPSS statistical software in the following model: Yijk = μ + αi + βj + (αβ)ij + εijk, in which Y is the analyzed variable; μ is the mean; αi is the effect of HMSeBA (i = 1 or 2); βj is the effect of LPS or H_2_O_2_ (j = 1 or 2); (αβ)ij refers to the interaction between treatment and LPS or H_2_O_2_ model; and εijk represents the error term. All results were plotted with GraphPad Prism 8.3 software. Data are shown as means ± standard errors (SE). *P* < 0.05 was considered statistically significant.

## Results

### Maternal 2-Hydroxy-4-Methylselenobutanoic Acid Supplementation During Gestation Increased the Expression of Selenoproteins in the Jejunum of Newborn and Weaning Piglets

**FIGURE 1:**
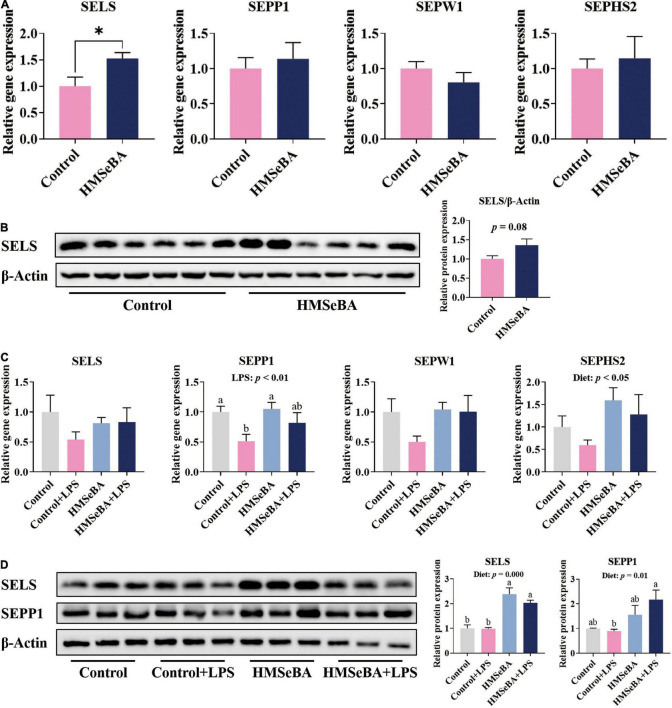
Effect of maternal HMSeBA supplementation during gestation on the expression of selenoprotein in the jejunum of piglets **(A)** Expression of selenoprotein genes in newborn piglets, *n* = 10 per group. **(B)** Relative protein abundance of SELS in newborn piglets, *n* = 6 per group. **(C)** Expression of selenoprotein genes in weaned piglets, *n* = 6 per group. **(D)** Relative protein abundance of SELS and SEPP1 in weaned piglets, *n* = 3 per group. Control, basal diet; HMSeBA, basal diet supplemented with HMSeBA, 0.3 mg Se/Kg of HMSeBA. + LPS, piglets challenged with LPS. Data are shown as mean ± SE. * *P* < 0.05. a, b Columns with different superscript letters mean significant differences (*P* < 0.05).

Maternal HMSeBA supplementation during gestation significantly increased the gene expression of SELS (Figure 1A) and had a tendency to increase the protein abundance of SELS in newborn piglets’ jejunum (Figure 1B). Besides, maternal HMSeBA supplementation upregulated the mRNA level of SEPHS2 (*P* < 0.05, Figure 1C) and protein levels of SELS and SEPP1 (*P* < 0.01, Figure 1D), while LPS administration reduced the mRNA level of SEPP1 in the jejunum of weaning piglets (*P* < 0.05, Figure 1C).

### Maternal 2-Hydroxy-4-Methylselenobutanoic Acid Supplementation During Gestation Reduced Endoplasmic Reticulum Stress in the Jejunum of Newborn and Weaning Piglets

**FIGURE 2:**
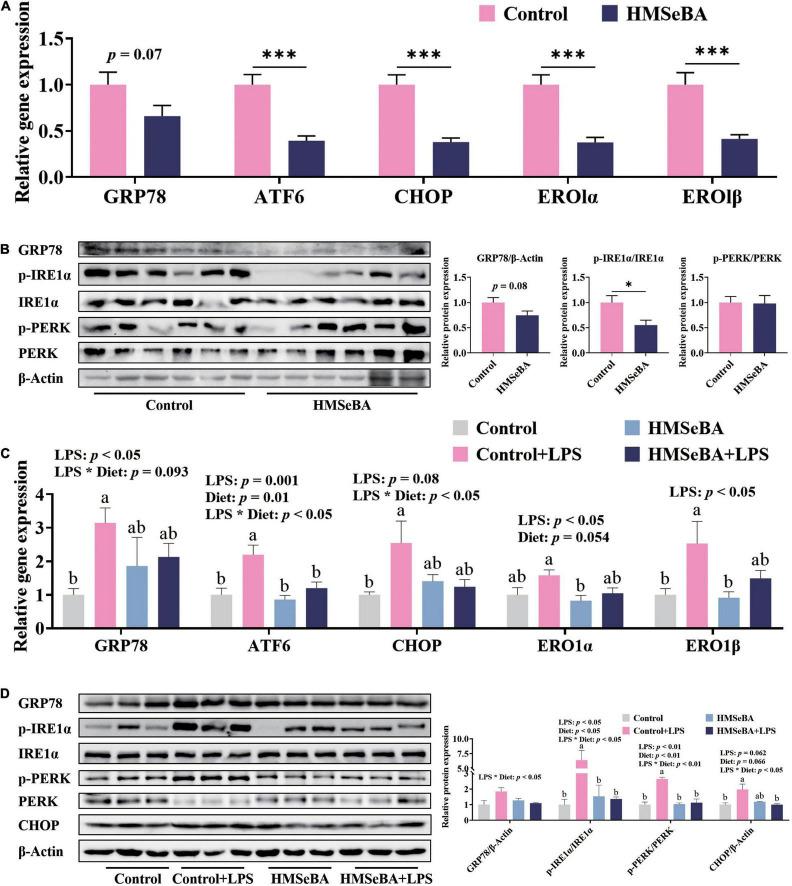
Effect of maternal HMSeBA supplementation during gestation on the expression of ER stress-related markers in the jejunum of piglets. **(A)** Gene expression of ER stress related markers in newborn piglets. *n* = 10 per group. **(B)** Expression of ER stress related proteins determined by western blot in newborn piglets. *n* = 6 per group. **(C)** Gene expression of ER stress related markers in weaned piglets. *n* = 6 per group. **(D)** Expression of ER stress related proteins determined by western blot in weaned piglets. *n* = 3 per group. Data are expressed as mean ± SE. * *P* < 0.05, *** *P* < 0.001. a,b Values with different superscript letters for same genes were significantly different (*P* < 0.05).

To assess whether maternal HMSeBA supplementation could regulate intestinal ER stress level of their offspring, we analyzed the ER stress related indexes in the jejunum of newborn piglets and LPS challenged weaned piglets. Results showed that the gene expression of ATF6, CHOP, ERO1α and ERO1β in the jejunum of newborn piglets were lower in HMSeBA group than those in the control group (*P* < 0.001, Figure 2A). Moreover, maternal HMSeBA supplementation markedly decreased the phosphorylation level of IRE1α compared with basal diet in newborn piglets’ jejunum (Figure 2B), while trended to decrease the mRNA and protein levels of GRP78 (Figures 2A,B).

In the jejunum of weaned piglets, LPS challenge significantly increased the gene expression of GRP78, ATF6, ERO1α and ERO1β, while maternal HMSeBA supplementation reversed the mRNA level of ATF6 that was increased by LPS (Figure 2C). What’s more, there were significant interactions between maternal diet and LPS on the gene expression of CHOP (Figure 2C). Besides, LPS administration markedly increased the phosphorylation levels of IRE1α and PERK in weaned piglets, while maternal HMSeBA supplementation reversed this effect of LPS (*P* < 0.05, Figure 2D). Furthermore, the interaction between maternal diet and LPS challenge significantly changed protein levels of GRP78, p-IRE1α/IRE1α, P-PERK/PERK and CHOP (*P* < 0.05, Figure 2D).

### 2-Hydroxy-4-Methylselenobutanoic Acid Treatment Upregulated the Expression of Selenoproteins and Improved the Antioxidative Capacity of Porcine Intestinal Epithelial Cells

**FIGURE 3:**
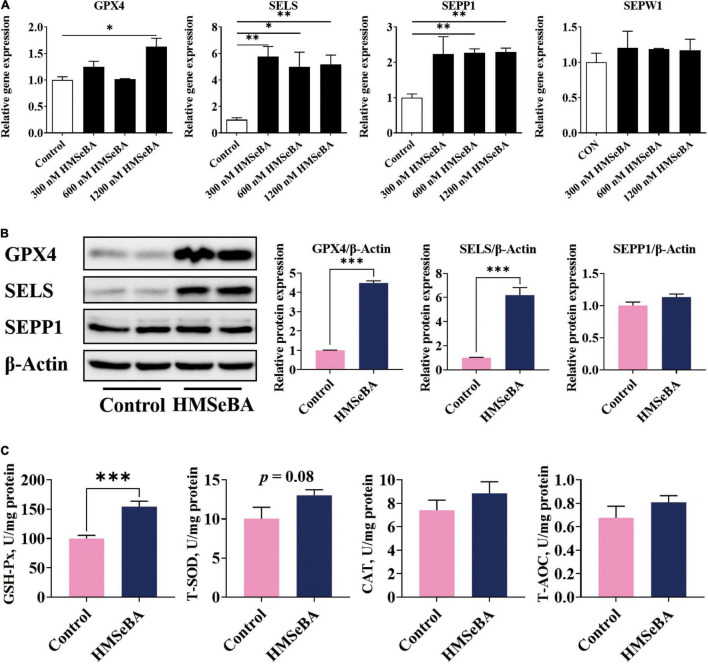
Effect of HMSeBA supplementation on the expression of selenoproteins and the antioxidative capacity in IPEC-J2 cells. IPEC-J2 cells were treated with 1.2 μM HMSeBA for 24 h. **(A)** Relative mRNA levels of selenoproteins. *n* = 3 for each group. **(B)** Relative protein abundance of GPX4, SELENOS and SELENOP. *n* = 4 for each group. **(C)** The activity of antioxidant related enzymes. *n* = 6 for each group. GSH-Px, glutathione peroxidase; T-SOD, total superoxide dismutase; CAT, catalase; T-AOC, total antioxidant capacity. Data are expressed as mean ± SE. * *P* < 0.05, ** *P* < 0.01, *** *P* < 0.001.

To assess whether HMSeBA treatment affects the expression of selenoproteins and the antioxidant capacity of IPEC-J2 cells, we detected the mRNA and protein levels of selenoprotein and activities of antioxidative markers. Results suggested that HMSeBA treatment increased the gene expression of GPX4, SELS, SEPP1 and SEPW1 in a dosage-dependent manner (*P* < 0.05, Figure 3A). Meanwhile, HMSeBA treatment substantially increased the protein abundance of GPX4 and SELS compared with control in IPEC-J2 cells (*P* < 0.001, Figure 3B). Besides, HMSeBA treatment improved the enzyme activity of GSH-Px (*P* < 0.001, Figure 3C) and tended to enhance the activity of T-SOD compared with control group in IPEC-J2 cells (*P* = 0.08, Figure 3C).

### 2-Hydroxy-4-Methylselenobutanoic Acid Treatment Reduced Reactive Oxygen Species Level and Endoplasmic Reticulum Stress in Hydrogen Peroxide Treated Porcine Intestinal Epithelial Cells

**FIGURE 4:**
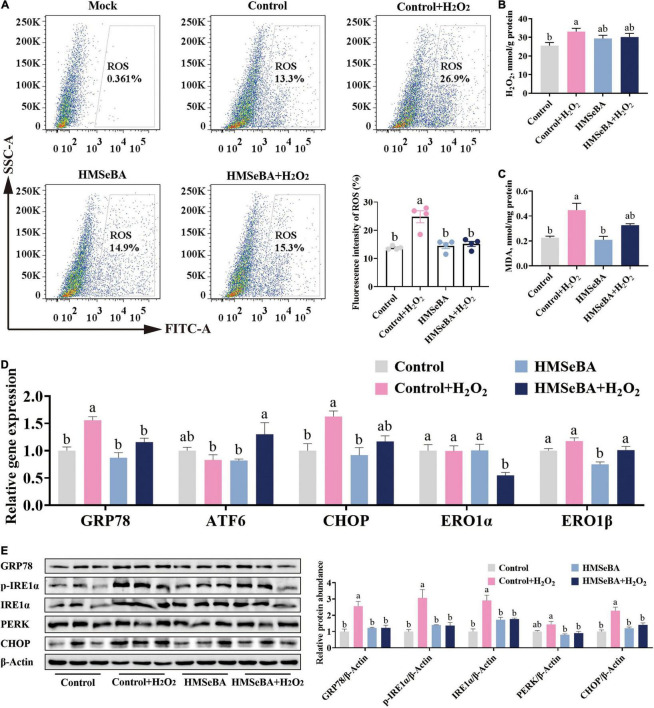
Effect of HMSeBA on hydrogen peroxide induced oxidative status and ER stress in IPEC-J2 cells. IPEC-J2 cells were pretreated with HMSeBA for 24 h, then co-treatment with H_2_O_2_ for 2 h. **(A)** ROS levels in IPEC-J2 cells. *n* = 4 for each group. **(B,C)** H_2_O_2_ and MDA levels in IPEC-J2 cells. *n* = 6 for each group. **(D)** Relative gene expression of ER stress related markers in IPEC-J2 cells. *n* = 4 for each group. **(E)** Protein levels of ER stress related markers in IPEC-J2 cells. *n* = 3 for each group. Mock, as a blank control; H_2_O_2_, hydrogen peroxide; MDA, malondialdehyde. Data are expressed as mean ± SE. a,b Values with different superscript letters for same genes were significantly different (*P* < 0.05).

To investigated whether HMSeBA can alleviate ROS induced ER stress, we established ER stress cell model with H_2_O_2_ treatment. The phosphorylation level of IRE1α was upregulated by H_2_O_2_ in a dosage-dependent treatment (Supplementary Figure 1A). Additionally, HMSeBA treatment drastically reduced the ROS level and MDA content induced by H_2_O_2_ compared with control group (*P* < 0.05, Figures 4A–C). Furthermore, H_2_O_2_ treatment markedly upregulated the mRNA level of GRP78 and CHOP, while HMSeBA reversed this effect of H_2_O_2_ (Figure 4D). Besides, H_2_O_2_ treatment increased the protein expression of GRP78, p-IRE1α, IRE1α and CHOP, and all of these could be reversed by HMSeBA treatment (*P* < 0.05, Figure 4E and Supplementary Figure 1B).

### Glutathione Peroxidase 4 and Selenoprotein S Were Involved in 2-Hydroxy-4-Methylselenobutanoic Acid Alleviating Reactive Oxygen Species Induced Endoplasmic Reticulum Stress in Porcine Intestinal Epithelial Cells

**FIGURE 5:**
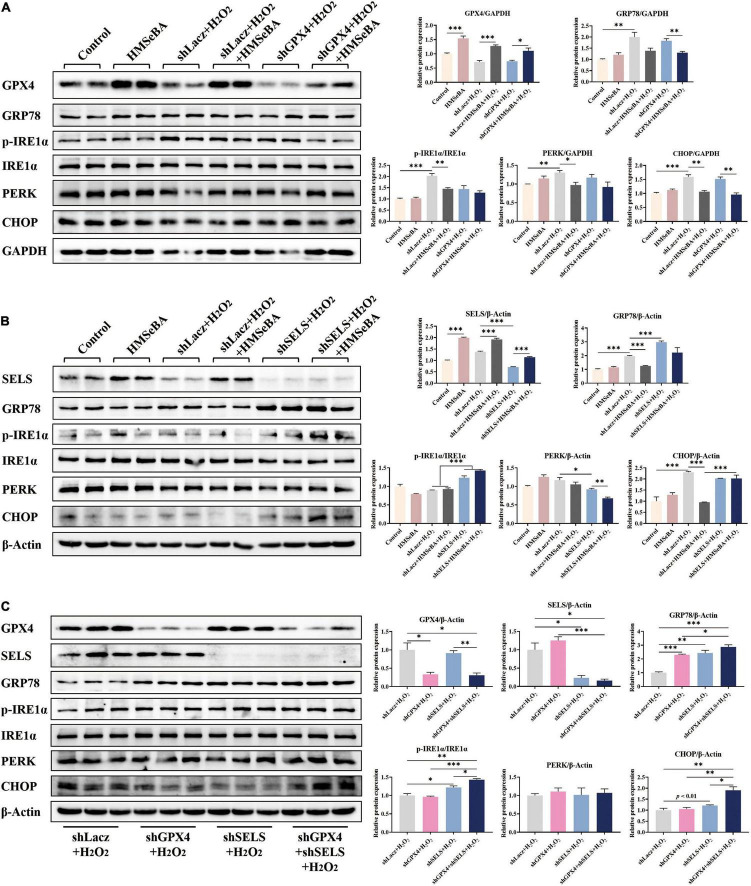
Roles of GPX4 and SELENOS in regulating ER stress induced by H_2_O_2_ in IPEC-J2 cells. **(A)** IPEC-J2 cells were infected with AdshGPX4, then were treated with H_2_O_2_ plus HMSeBA. Protein levels of ER stress related markers were detected. **(B)** IPEC-J2 cells were infected with AdshSELS, then were treated with H_2_O_2_ plus HMSeBA. Protein levels of ER stress related markers were detected. **(C)** IPEC-J2 cells were infected with AdshGPX4 or/and AdshSELS, then were treated with H_2_O_2_. Protein levels of ER stress related markers were detected *n* = 4 or 3 for western blot assay. Data are expressed as mean ± SE. * *P* < 0.05, ** *P* < 0.01, *** *P* < 0.001.

In order to elucidate the role of GPX4 and SELS in regulating ER stress, we performed gene knockdown study with GPX4 and SELS in IPEC-J2 cells. Results showed that HMSeBA upregulated the protein level of GPX4 in IPEC-J2 cells, while AdshGPX4 significantly decreased the protein abundance of GPX4 compared with control group, under both basal or HMSeBA treatment conditions (Figure 5A). HMSeBA treatment reduced the phosphorylation level IRE1α and the protein level of CHOP, while GPX4 knockdown blocked the reduce effect of HMSeBA on IRE1α phosphorylation (*P* < 0.05, Figure 5A).

Analogously, HMSeBA upregulated the protein level of SELS in IPEC-J2 cells, while infection of AdshSELS almost eliminated this effect of HMSeBA on SELS (Figure 5B). Knockdown of GPX4 reversed the reducing effect of HMSeBA on the protein levels of GRP78 and CHOP (*P* < 0.05, Figure 5B). Knockdown of SELS furtherly upregulated the protein levels of GRP78 and phosphorylation level of IRE1α (*P* < 0.05, Figure 5B).

Knockdown of both GPX4 and SELS increased the protein level of GRP78, compared with control group or GPX4 knockdown (*P* < 0.05, Figure 5C), while induced the phosphorylation level of IRE1α and protein level of CHOP compared with control group or alone knockdown (*P* < 0.05, Figure 5C).

## Discussion

Endoplasmic reticulum (ER) stress induced by LPS or ROS contributes to intestinal inflammation diseases via disturbing the intestinal redox homeostasis (10, 23, 24), while selenium or selenoproteins are reported to be vital for sustaining the intestinal redox homeostasis (25). In our previously studies, maternal HMSeBA supplementation decreased the inflammation and autophagy levels in ileum and ER stress signal in thymus and spleen of their offspring all by improving the expression of selenoproteins (16, 20). Here, we also found that maternal HMSeBA supplementation increased the protein abundance of SELS and inhibited the ER stress signal induced by LPS in piglets’ jejunum. Additionally, HMSeBA treatment upregulated the expression of GPX4 and SELS, while significantly reduced the ROS level and intensity of ER stress signal induced by H_2_O_2_ in IPEC-J2 cells. To our knowledge, this is the first time of the founding of that maternal HMSeBA supplementation regulating ER stress signal of the offspring’ intestine, what’s more, synergistic regulations of GPX4 and SELS can also protect IPEC-J2 cells against ER stress *in vitro*.

Selenoproteins play indelible roles in the process of life and diseases, and most selenoproteins are central sites for regulating redox homeostasis, such as GPX, SEPP1 and ER resident-selenoprotein SELS (26). SELS is involved in ER homeostasis regulation by forming a complex with ubiquitin ligase E3, Derlin-1, selenoprotein K and p97ATPase (27). Du et al. found that SELS could protect HepG2 cells against apoptosis induced by ER stress (28). Additionally, SEPP1 was also involved in palmitic acid induced ER stress (29). These findings suggested that SELS and SEPP1 may play important roles in ER stress regulation. In our study, maternal HMSeBA supplementation increased the expression of SELS and SEPP1 of their progeny, this provided the material basis for maternal HMSeBA to regulate the intestinal ER stress signal of their offspring.

Piglets are susceptible to oxidative stress during birth and weaning, and oxidative stress can induce ER stress (30, 31). To further explore the effect of maternal HMSeBA supplementation on intestinal ER stress signal of their offspring, we detected ER stress-related markers in newborn and weaned piglets’ jejunum. Meanwhile, we also established LPS-induced ER stress model in weaned piglets to further determine the protective effect of HMSeBA. Our results suggested that maternal HMSeBA supplementation reduced the phosphorylation level of IRE1α and gene expression of ATF6, CHOP and their downstream targets ERO1α and ERO1β in jejunum of newborn piglets. Analogously, maternal HMSeBA supplementation also suppressed the activation of IRE1α/PERK/ATF6-CHOP signaling pathway in jejunum of weaned piglets after LPS challenge. Activation of intestinal ER stress signal may cause multiple adverse outcomes, such as barrier disruption and inflammation (32). Jiang et al. proved that GRP78 was involved in ER stress associated apoptosis induced by LPS (10). IRE1α was the endogenous substrate of ER-associated degradation (33), otherwise, the IRE1-RIDD-RIG-I pathway was considered as an innate immune signal which located at mucosal surfaces (14). Furthermore, Lu et al. concluded that heat-labile enterotoxin caused apoptosis of IPEC-J2 cells via PERK-CHOP pathway (24). Therefore, maternal HMSeBA supplementation inhibited the ER stress signal induced by oxidative stress or LPS via enhancing the expression of SEPP1 and SELS.

In order to further explore the specific mechanism of HMSeBA regulating intestinal ER stress signal, we established an ERS model of IPEC-J2 cells *in vitro*. For a long time, hydrogen peroxide (H_2_O_2_) was considered as a classic model for oxidative stress, and it can also activate ER stress through modulation of ROS generation (9). In addition, exposure of HepG2 (human hepatoblastoma cell line) to H_2_O_2_ has been demonstrated that would increase ROS levels and induce ER stress in a dosage-dependent manner (34). This is consistent with our result, the protein abundance of p-IRE1α increased with a dosage-dependent upregulation of H_2_O_2_. On the other hand, previous study have found that cistanche deserticola protected myocardial cell from ER stress related apoptosis through suppressing H_2_O_2_-induced ERS (35). In addition, N-acetylcysteineould could inhibit ER stress induced by ROS products of porcine epidemic diarrhea virus via GRP78/IRE1pathway (36). All these findings suggested that antioxidants may inhibit ER stress signal induced by ROS. In our present study, HMSeBA treatment increased the gene expression of GPX4, SELS, SEPP1 and the protein abundance of GPX4 and SELS in IPEC-J2 cells, at the same time, HMSeBA attenuated the ER stress signal induced by H_2_O_2_.

Selenoprotein S (SELS) is the selenoprotein which mainly played roles in reflecting the UPR status and involved ER homeostasis regulation (37), while GPX4 is one of the selenoproteins which can effectively protect the normal physiological function of ER by inhibiting the production of lipid peroxidation (38). To explore whether GPX4 or SELS target in regulating ER stress, we knocked down GPX4 and SELS separately or together. Results showed that knockdown of SELS seems to more significantly up-regulate the H_2_O_2_-induced ER stress compared with knocking down GPX4. In addition, knockdown of GPX4 and SELS together showed a stronger ER stress signal than knocking down one of them alone. Therefore, we suspect that SELS, which located in ER, plays a major role in regulating ER stress. What’s more, the deficiency of GPX4 did not enhance the ER stress signal induced by H_2_O_2_, while in the absence of SELS, the lack of GPX4 would cause further deterioration of the ER stress signal. In other words, GPX4 can cooperate with SELS to participate in the regulation of H_2_O_2_-induced ER stress signal. Therefore, HMSeBA is involved in alleviation of H_2_O_2_-induced ER stress through reducing generation of ROS by increasing the expression of GPX4 and SELS.

**FIGURE 6:**
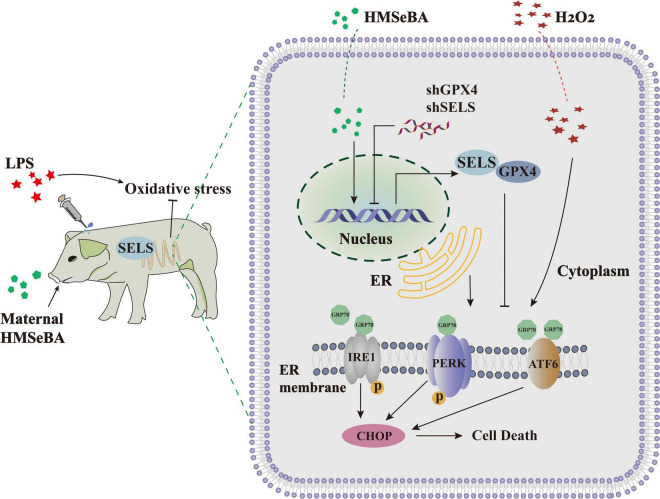
General view of the effect of HMSeBA on intestinal ER stress. A model for HMSeBA supplementation regulates ER stress induced by LPS or H_2_O_2_ *in vivo* and *vitro*. Only core components of the pathway are shown. LPS or H_2_O_2_ administration induce ER stress through activating the GRP78-IRE1α-CHOP signaling pathway. While HMSeBA treatment improves the expression of GPX4 and SELS, thereby suppresses the ER stress signal, ameliorates the redox status. Maternal HMSeBA is involved in regulating the ER stress signal and health of intestine.

In conclusion, our results showed that maternal HMSeBA supplementation could relieve intestinal ER stress in piglets by enhancing the expression of GPX4 and SELS (Figure 6). This study provides new information about functions of HMSeBA in the intestine, identifies new targets for HMSeBA regulating ER stress, and may provide a strong basis for organic selenium application on protecting the intestine against ER stress-related intestinal diseases.

## Data Availability Statement

The original contributions presented in the study are included in the article/Supplementary Material, further inquiries can be directed to the corresponding authors.

## Ethics Statement

The animal study was reviewed and approved by Institutional Animal Care and Use Committee of the Laboratory Animal Center at Sichuan Agricultural University (SICAU-2015-033).

## Author Contributions

BF, DD, and DM designed the study. DD and HZ conducted the research. XJ, LC, ZF, SX, YL, YZh, JL, CH, YZo, and LL analyzed the data. DD and DM wrote the manuscript. BF and DW revised the manuscript. BF had primary responsibility for the final contents. All authors read and approved the final manuscript.

## Conflict of Interest

The authors declare that the research was conducted in the absence of any commercial or financial relationships that could be construed as a potential conflict of interest.

## Publisher’s Note

All claims expressed in this article are solely those of the authors and do not necessarily represent those of their affiliated organizations, or those of the publisher, the editors and the reviewers. Any product that may be evaluated in this article, or claim that may be made by its manufacturer, is not guaranteed or endorsed by the publisher.
