# Supplementary material for: Maternal Organic Selenium Supplementation Relieves Intestinal Endoplasmic Reticulum Stress in Piglets by Enhancing the Expression of Glutathione Peroxidase 4 and Selenoprotein S
Source: Front Nutr. 2022 May 6;9:900421. doi: 10.3389/fnut.2022.900421 (PMC9121856; doi:10.3389/fnut.2022.900421)
Supplement: Supplementary file 1 [file Data_Sheet_1.docx]

**Table S1.** Primer sequences used for real-time PCR.

| **Genes** | | **Primer** | **Sequence (5’→3’)** | **Accession no.** |
| --- | --- | --- | --- | --- |
| GPX4 | Forward | | TGAGGCAAGACGGAGGTAAACT | NM_214407 |
|  | Reverse | | TCCGTAAACCACACTCAGCATATC |  |
| SELS | Forward | | GAGGCAGAGGCACCTGGAT | NM_001164113.1 |
|  | Reverse | | CTGCTAAAGCCTCCTGTCGTTT |  |
| SEPP1 | Forward | | AACCAGAAGCGCCAGACACT | EF113596 |
|  | Reverse | | TGCTGGCATATCTCAGTTCTCAGA |  |
| SEPW1 | Forward | | CACCCCTGTCTCCCTGCAT | NM_213977 |
|  | Reverse | | GAGCAGGATCACCCCAAACA |  |
| SEPHS2 | Forward | | TGGCTTGATGCACACGTTTAA | EF033624 |
|  | Reverse | | TGCGAGTGTCCCAGAATGC |  |
| GRP78 | Forward | | GGTAAGTGGGGTTGGTGGAA | XM_02106883 |
|  | Reverse | | CACGGCCATTCTTGAACACC |  |
| ATF6 | Forward | | AGGGAAAGATTCCACTTGGTCT | XM_02108951 |
|  | Reverse | | GCTGCAGGTCTTCAGTGTCT |  |
| CHOP | Forward | | GCTGGAAAGCAACGCATGAA | NM_00114484 |
|  | Reverse | | ACCATCCGGTCAATCAGAGC |  |
| ERO1α | Forward | | AGGCTGTTCTTCAGTGGACC | NM_00113762 |
|  | Reverse | | TCTGGTCCCTTGTAACCCGT |  |
| ERO1β | Forward | | TCACCGGAGTCCTGGATGAT | XM_01398162 |
|  | Reverse | | GAAAGGACAGGGTCGCTTCA |  |
| β-Actin | Forward | | AACTGGAACGGTGAAGGTGA | AY550069.1 |
|  | Reverse | | CTTTTGGAAAGGCAGGGACT |  |

GPX4, glutathione peroxidase 4; SELS, selenoprotein S; SEPP1, selenoprotein P; SEPW1, selenoprotein W; SEPHS2, selenophosphate synthetase 2; GRP78, glucose-regulated protein 78; ATF6, activating transcription factor 6; CHOP, C/EBP-homologous protein; ERO1α, endoplasmic reticulum oxidoreductase 1 alpha; ERO1β, endoplasmic reticulum oxidoreductase 1 beta; β-actin, beta-actin.


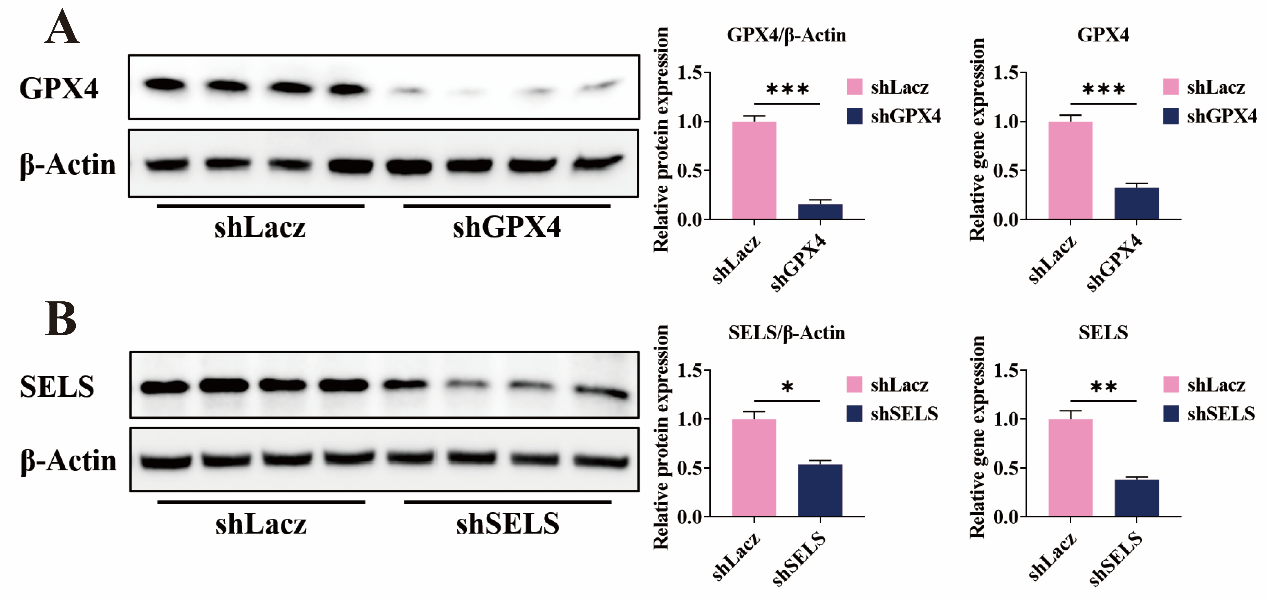


**Figure S1.** Knockdown efficiency of shGPX4 and shSELS in IPEC-J2 cells. (A) Knockdown efficiency of mRNA level and protein abundance of shGPX4 in IPEC-J2 cells. (B) Knockdown efficiency of mRNA level and protein abundance of shSELS in IPEC-J2 cells. n = 4 for western blot assay and RT-PCR administration. Data are expressed as mean ± SE. * *P* < 0.05, ** *P* < 0.01, *** *P* < 0.001.


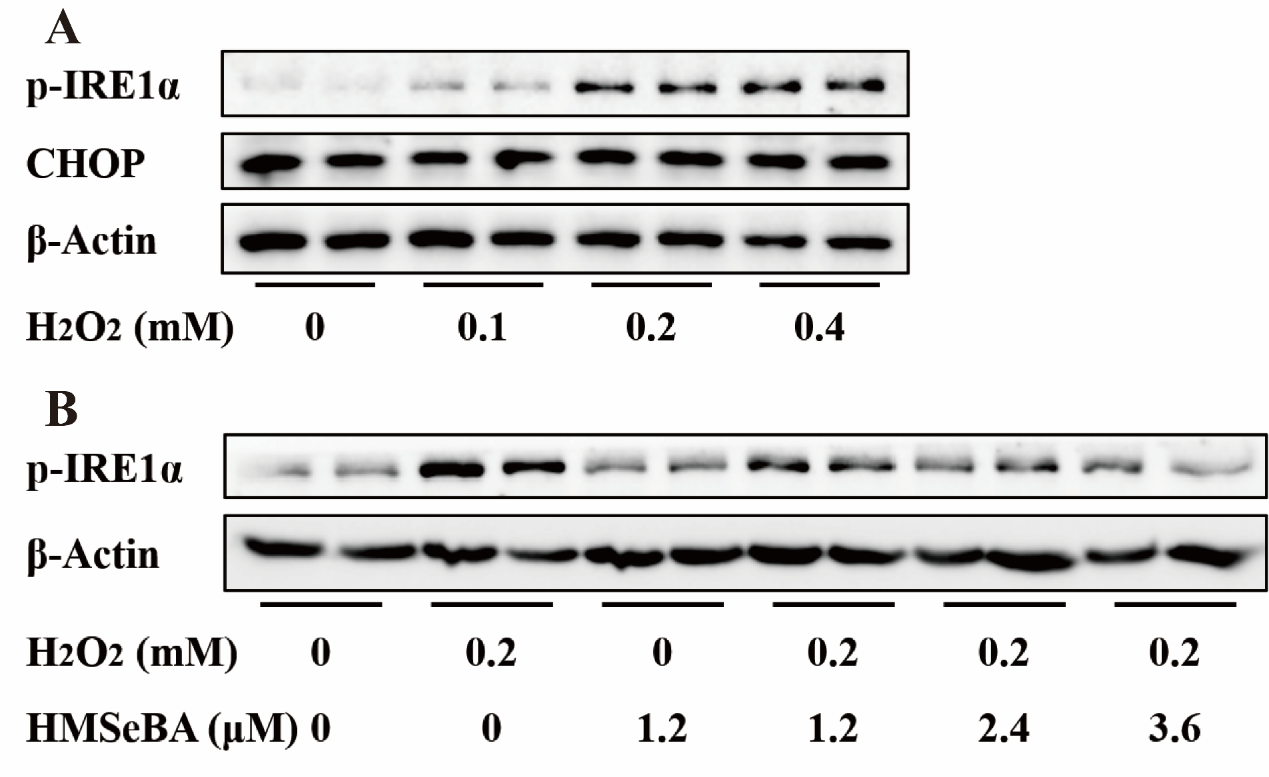


**Figure S2.** (A) Effect of H_2_O_2_ treatment on the expression of ER stress-related markers in IPEC-J2 cells. H_2_O_2_ administration for 2 hours. (B) HMSeBA treatment reduced the protein abundance of p-IRE1α induced by H_2_O_2_ in IPEC-J2 cells. H_2_O_2_ administration for 2 hours, HMSeBA pretreatment for 24 hours.
